# Interventions Focused by Nurses for Reducing Negative Effect of Traumatic Experience on Victims of Sexual Violence: A Scoping Review

**DOI:** 10.3390/healthcare11010125

**Published:** 2022-12-31

**Authors:** Iyus Yosep, Rohman Hikmat, Ai Mardhiyah, Mamat Lukman

**Affiliations:** 1 Department of Mental Health, Faculty of Nursing, Universitas Padjadjaran, Bandung 40132, Indonesia; 2 Professional Nursing Program, Faculty of Nursing, Universitas Padjadjaran, Bandung 40132, Indonesia; 3 Department of Pediatric Nursing, Faculty of Nursing, Universitas Padjadjaran, Bandung 40132, Indonesia; 4 Department of Community Nursing, Faculty of Nursing, Universitas Padjadjaran, Bandung 40132, Indonesia

**Keywords:** sexual violence, traumatic experience, nursing interventions

## Abstract

Sexual violence has increased quite rapidly. Sexual violence can be in the form of physical or verbal violence. The impact of sexual violence causes a traumatic experience that causes physical problems, psychological problems, loss of the future, and causes the risk of death. Nurses have an important role in reducing the impact of sexual violence on victims. The purpose of this study is to describe nursing interventions to reduce the impact of traumatic experiences experienced by victims of sexual violence. This study used a scoping review method. The literature used in this study is from CINAHL, PubMed, and Scopus. Keywords in this study are sexual violence, traumatic experience, impact, and victims. Search strategy used PRISMA Extension for Scoping Reviews to select articles for this study. The inclusion criteria were that the sample was female victims of sexual violence, studies employed a randomized control trial or quasi-experimental research design, and the publication period was of the last 10 years (2013–2022). We found 10 articles that matched the inclusion and exclusion criteria. The findings from this scoping review show that nursing interventions can reduce the impact of traumatic experiences on victims of sexual violence. There are three methods of nursing intervention, namely cognitive behavior, counseling, and web-based intervention. The samples are from developing and developed countries. The range of the samples are from 35–1250 respondents. Nursing interventions focus on victims in order to improve mental health and reduce the traumatic impact experienced by victims of sexual violence. The activities carried out were psychoeducation, keeping a daily journal, and discussions related to the traumatic experiences experienced. Nurses as health workers have a role to provide comprehensive nursing care to victims of sexual violence by taking into account the characteristics and impact of trauma experienced by victims of sexual violence.

## 1. Introduction

Cases of sexual violence are now increasing every year. Physical and/or sexual violence against women in 2021 is 26.1%. According to a report by the United Nations Emergency Children’s Fund (UNICEF), in 28 European countries, there are 2.5 million young women who reported having experienced sexual harassment, either through physical contact or not before the age of 15 years [1]. The WHO estimates that in 2017, there are around 1 billion children under the age of 2–17 years who have experienced physical, emotional, and sexual violence [2]. In detail, as many as 13.8% of women aged 15–64 years during their lifetime experienced physical violence in 2021 [3]. The prevalence of violence against women is 3.02% of 10,000 adolescents in developing countries [4]. 

Sexual violence is any act that appears in the form of coercion or threatening to have sexual intercourse (sexual intercourse), performs torture or acts sadistically, and leaves someone, including those who are classified as children, after having sexual relations [5]. All behavior that leads to acts of sexual harassment against children both at school, in the family, or in the environment around the child’s residence is also included in the category of violence or a violation of this type of children’s rights [6,7]. Cases of child rape, sexual abuse perpetrated by teachers, other people, and even stepparents who are often exposed in various mass media reports are concrete examples of this form of violence [8,9,10].

The negative effect of sexual violence are physical problems and psychological problems. The results of previous research explain that the impact of violence experienced by children who are victims of sexual violence, among others, are that they do not want to go to school and have feelings of low self-esteem, they withdraw from society, and are easily offended and aggressive [7,11,12]. Other studies explain that children who get cruel treatment will become aggressive, and they will in turn become aggressive adults as well [13,14]. Incidence of violence against children will generally be recorded in the child’s subconscious, and will be carried over to adulthood and even throughout his life [15].

Psychological reactions that appear immediately after individuals’ experience with sexual violence include shock, disbelief, denial, feeling afraid, confused, anxious, and withdrawn [4,16]. Another psychological impact is that women victims of violence often feel low or decreased self-esteem, feel guilty, ashamed, experience sleep disturbances, symptoms of post-traumatic stress disorder, and sexual problems [17,18]. Sexual violence greatly impacts how victims perceive their world [19]. Generally, victims of sexual violence will experience changes in beliefs about security, power, trust, and intimacy [20]. Risky behavior is also generally carried out by individuals as a form of coping against the unbearable effects of victimization [21].

Based on the description above, it can be seen that trauma is one of the psychological impacts that is quite serious for victims, not only in the short term but can also last for a long time and cannot be predicted. Treatment of trauma to victims of sexual violence can be done by nurses through several methods [2,22]. Some victims of sexual violence who experience trauma develop coping strategies that can reduce or suppress negative feelings [23]. The behavioral approach is also effectively applied to reduce the traumatic symptoms experienced by victims of sexual violence [24].

The impact of sexual violence on the victim can have a traumatic effect that can cause major impacts such as a loss of the future and the risk of suicide. Nurses as health workers need to deal seriously with the impact experienced by victims. However, not all nurses understand the methods that can be used in dealing with victims of sexual violence that have a traumatic impact. So, a study is needed that discusses nursing intervention methods that can be done to reduce the impact of traumatic experience, experienced by victims of sexual violence.

## 2. Methods

### 2.1. Design

This study used the design scoping review. Scoping review is a method that can discuss hot topics that are currently developing. The authors chose this design study because it can cover a wide conceptual range based on the objectives of this study. The steps taken in this study were designing research questions, creating an article search strategy by determining keywords, selecting articles for review, analyzing and describing the results of the scoping review, and preparing a report on the results of this study. The article search strategy in this study used PRISMA Extension for Scoping Reviews (PRISMA-ScR) to select and identify various topics that discuss nursing interventions to reduce the impact of traumatic experiences, experienced by victims of sexual violence.

### 2.2. Search Methods

This study conducted an article search through three databases, namely CINAHL, Pubmed, and Scopus. These databases were the largest and most extensive databases of abstracts and citations of peer-reviewed literature: scientific journals, books, and conference proceedings. The keywords used in English were: “nursing care OR nursing intervention” AND “traumatic experience effect OR traumatic effect” AND “victim” AND “sexual violence OR sexual aggression”. The research questions are: What types and how can nursing interventions reduce the impact of traumatic experience on victims of sexual violence?

### 2.3. Inclusion and Exclusion Criteria

**Figure 1:**
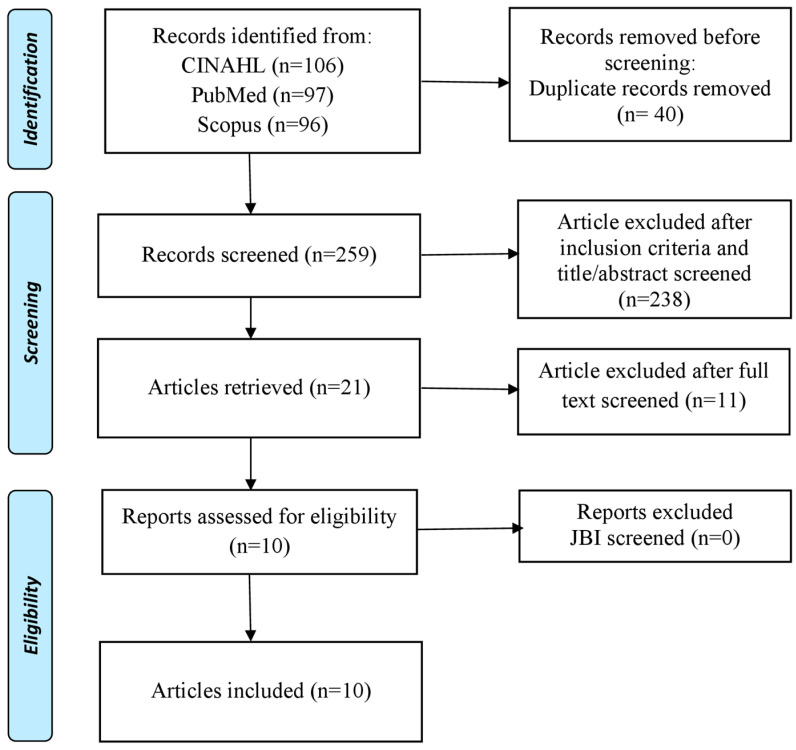
PRISMA Flow Diagram.

The search strategy and article selection used PRISMA Extension for Scoping Review (PRISM -ScR) to identify types of nursing interventions that can reduce the impact of traumatic experiences on victims of sexual violence (Figure 1). The inclusion and exclusion criteria for finding articles that are appropriate for this study are samples of victims of sexual violence, using English, full text, nursing interventions, design randomized control trials or quasi-experiments, articles that are original research, and the time setting of the last 10 years (2013–2022). While the exclusion criteria in this study were that the sample was not a victim of sexual violence and had no traumatic experience.

### 2.4. Data Extraction

Data extraction in this study uses manual tables. Manual tables can contain various information needed to describe the results of studies that have been carried out. This table can also be material for writers in making discussion points to make them more comprehensive and focused and make it easier for writers to classify data. The contents of the articles written in the extraction table are the author, year, country, research design, population and sample, procedures, interventions, and results of the study.

### 2.5. Quality Appraisal

Quality appraisal is needed in this study to assess the quality of the articles. The quality of the articles was seen by the authors using The Joanna Brigs Institute (JBI) tools. JBI contains statements based on research design to assess the quality of articles. The statement is given a score consisting of yes, no, unclear, and not applicable. Score 1 is for the yes score and score 0 is for the other scores. The author determines the standard quality of articles based on the JBI assessment, which is above 75% for use in this study. All authors double checked to ensure that the articles obtained are relevant to the purpose of this study.

### 2.6. Data Analysis

The data analysis used a descriptive approach. Data analysis begins with classifying the data obtained based on the study results, then the author describes the study results based on the articles reviewed. All authors reviewed the articles obtained twice to ensure the data obtained were correct. Data analysis is seen from the manual tables that have been made by the authors to see nursing intervention methods for victims of sexual violence and how the effectiveness of interventions is to reduce the impact of traumatic experience. After obtaining the data, all authors analyzed and explained them based on the results of the study.

## 3. Results

The number of articles obtained from the search database is 299 articles. After duplicating the collected articles, 259 articles were obtained. Furthermore, after elimination based on the inclusion criteria and checking the title and abstract, 21 articles were found. We read the full text, and we found 10 articles to be analyzed. We used the JBI Critical Appraisal Tool assessment method to analyze the quality of articles, and all authors gave a standard score of JBI to the articles to be analyzed as above 75% based on criteria and topic relevance (Table 1).

There were 10 articles that discussed nursing interventions to reduce the effects of traumatic experiences on victims of sexual violence. The research locations in this scoping review range from developed and developing countries. The sample in this study is in the range of 35–1250 respondents. Respondents mostly include women and are still teenagers. We have read and analyzed 10 articles, and we classified the intervention with three programs; these were cognitive behavior, counseling, and web-based intervention. 

### 3.1. Cognitive Behavior Therapy on Victims of Sexual Violence

Trauma-Based Cognitive Therapy is a cognitive behavior intervention that utilizes modules in its intervention. The module contains guidance on interactivity, didactic activities, trauma recovery, and problem solving. This module runs over 14 sessions over a period of 14 weeks. Imagery Rehearsal Therapy is one of the cognitive behavior interventions carried out for 12 weeks. The intervention consisted of accepting the traumatic event, health education, role playing, and keeping a daily journal to make the time more productive. The results of the study show that both interventions can reduce the impact of sexual violence.

The other two interventions are narrative exposure therapy and Cognitive Behavior Therapy. Both of these interventions were carried out for 8–10 weeks with the intervention having 8–12 sessions. These two interventions are part of cognitive behavior therapy that utilizes psychoeducation, relaxation skills, affective attention, and cognitive coping. The results of the intervention showed that there was a decrease in the negative impact of victims who experienced sexual violence.

### 3.2. Counseling Therapy

This study found four counseling programs in reducing the impact of traumatic events on victims of sexual violence. The four interventions are psychosocial interventions, Trauma Recovery Program, Common Elements Treatment Approach, and Trauma-Informed Support, Skills, and Psychoeducation Intervention. Of the four interventions, all were carried out in a time span of 15–18 weeks and 8–12 sessions. Each intervention was carried out in groups and individually. In the group intervention, general information about sexual violence was discussed so that participants have the same perception related to sexual violence. Then, the intervention carried out individually is an intervention that focuses on accepting and solving the problems encountered.

Counseling intervention has the aim of building adaptive coping in dealing with stressors in the form of traumatic events due to sexual violence. Participants were also trained on self-confidence about their condition so that they can be productive again in carrying out their lives. The results of the intervention showed that there was a significant improvement in the quality of life for victims of sexual violence.

### 3.3. Web-Based Intervention

Web-Based Sexual Violence Bystander Intervention and web-based weWomen/ourCircle intervention are two types of nursing interventions in reducing the impact of online sexual violence. This intervention consists of psychoeducation, meditation, problem recognition, and problem solving. Participants are also given space to tell the facilitator stories outside of the predetermined agenda. This intervention was carried out for 12–15 weeks. Any material provided via video can be played repeatedly. Providing Zoom services for asynchronous discussions can also increase the enthusiasm and motivation of participants in carrying out interventions. The results of this intervention study show that there is an increase in the quality of life and a decrease in depressive symptoms in victims of sexual violence.

The results of the analysis of the articles are presented in manual form from all authors presented as follow (Table 2):

## 4. Discussion

The results of this scoping review show that nursing interventions can reduce the negative impact of traumatic experiences due to sexual violence. The author classified the results of the study into three nursing intervention methods, namely cognitive behavior, counseling, and web-based intervention. Each intervention is carried out by facilitators, namely nurses and other health workers such as psychologists. The interventions taken in this study are from the last 10 years to obtain the latest types of interventions in reducing the impact of traumatic experiences due to sexual violence.

Based on study results, sexual violence often occurs among women. Previous studies have shown that 80% of incidents of sexual violence occur among women [32]. The cause of the high rate of sexual violence that occurs among women is caused by several factors [29,33,34]. This happens because there is gender inequality between men and women in society, and it has become a culture that men are considered superior and women are considered inferior [35,36]. Some men think that power and violence are a form used to control other people. Another studies shows that men who are perpetrators of sexual violence think that they are superior to women [4,37].

The interventions in this study were carried out in developed and developing countries. Incidents of sexual violence can occur anywhere, including developed and developing countries. This is in line with previous studies which show that sexual violence often occurs in developing countries [38]. Meanwhile, other studies show that sexual violence occurs in free countries, namely developed countries [39,40]. So, the interventions in this study were quite diverse because they were carried out in developed and developing countries.

The intervention in this study was carried out over a period of 8–15 weeks. This long time is used to recover from the trauma experienced by victims of sexual violence. Traumatic disorders make it difficult for victims of sexual violence to concentrate, have difficulty communicating, and have cognitive impairments. Psychological problems occurred for a long time (2 months) [41,42]. So, the process of reducing mental health problems due to traumatic events of sexual violence takes 2–5 months [8,43]. In another study conducted to deal with trauma in victims of sexual violence, it took 3 months [35]. This is in line with previous studies that conducted interventions for acceptance of traumatic events due to sexual violence for 2 months [2,44]. Intensive care is required for victims of sexual violence so that there is acceptance and healing from the traumatic effects. 

The method of nursing intervention that can be done is cognitive behavior. Cognitive behavior is a form of psychotherapy that aims to improve your thought process (cognitive) and behavior. The results of previous studies show that cognitive behavioral therapy is a form of psychotherapy that has been shown to be effective for a variety of problems, including depression, anxiety disorders, alcohol and substance abuse problems [45], family problems, eating disorders, and severe mental illness [46]. Cognitive therapy can improve the cognitive abilities of victims of sexual violence in dealing with traumatic events and solving the problems they face [47]. Cognitive behavioral therapy has also been shown to be effective in reducing the traumatic effects on victims who have experienced violence. 

Another method that can be used to reduce the negative impact of traumatic experience on victims of sexual violence is counseling. Counseling is carried out by a psychologist or psychiatric nurse as a facilitator. Counseling has an important role in accepting traumatic events experienced by previous victims [31]. The facilitator can provide opportunities for victims to express violence that has been experienced before [48]. Victims will also be invited to discuss ways to find solutions to the problems faced by victims. Counseling is carried out by increasing the participant’s ability to express feelings and build trust in others to heal their trauma. Other studies also show that counseling can improve mental health in victims who experience violence [49,50]. Victims of violence will be given space without judgment from the facilitator who actively listens to the experiences of sexual violence experienced by victims.

Web-based intervention is an intervention that can be done online for victims of sexual violence. This intervention utilizes technology to provide education, training, and discussion to victims of sexual violence. Web-based interventions can reach every individual without being limited by distance and time. So, this intervention process is more affordable and more efficient [10]; this is in line with previous studies showing that web-based interventions can reduce the impact of traumatic experiences on victims of sexual violence [10,51]. Victims of sexual violence are also given training to deal with stress and depression that arise as a result of the traumatic events experienced by the victims [42]. The impact of sexual violence experienced by victims can be significantly reduced through interventions made via the web.

The impact of sexual violence can lead to a decrease in the victim’s mental health. Health workers have a role to play in reducing the negative events and impacts resulting from traumatic experiences, experienced by victims of sexual violence. In addition to the role of health workers, the government also needs to make policies to provide a safe space for every individual and avoid sexual violence.

### Limitations

The limitation in this study is that the literature reviewed is limited to the last 10 years (2013–2022), so it cannot discuss literature beyond that period; thus, this study cannot comprehensively discuss interventions. In addition, this study also has further limitations as the design is limited to randomized control trials and quasi-experiments; thus, this study cannot discuss interventions carried out without using this design. These limitations are carried out by the author in order to obtain relevant and up-to-date data sources so that they can be carried out in the present.

## 5. Conclusions

This study showed that there are 10 articles from three databases that discuss nursing interventions in reducing the impact of traumatic experiences on victims of sexual violence. The methods used in the nursing interventions are cognitive behavior, counseling, and web-based intervention. Activities carried out in the form of psychoeducation, physical activity, meditation for relaxation, daily journals, and problem solving discussions. Nurses have an important role to be facilitators, educators, and counselors in every activity carried out. The implication of this study is that there is a basis for nurses to intervene in victims of sexual violence to reduce the impact of the traumatic experience they experience. In addition, this intervention can also be used as material in making policies on sexual violence by the government and health services. Suggestion for future research are the need for research on the impact of sexual violence experienced by women and the effect of the nursing interventions by women nurses to reduce the negative effect of sexual violence on women with victims of sexual violence.

## Figures and Tables

**Table 1:** JBI Critical Appraisal Tool.

Author, Pubslished Year	JBI Critical Appraisal Tool	Study Design
[25]	84.6% (11/13)	RCT
[26]	92.3% (12/13)	RCT
[26]	76.9% (10/13)	RCT
[27]	76.9% (10/13)	RCT
[10]	92.3% (12/13)	RCT
[28]	84.6% (11/13)	RCT
[9]	84.6% (11/13)	RCT
[29]	92.3% (12/13)	RCT
[30]	76.9% (10/13)	RCT
[31]	76.9% (10/13)	RCT

**Table 2:** Extraction data.

No	Author and Year	Purpose	Country	Method	Sample	Intervention	Result
1.	[25]	Reduce traumatic experience and improve quality of life	Spain	RCT	50 students	Trauma-Based Cognitive Therapy	Significantly reduce traumatic stress and improve quality of life
2.	[26]	Reduce effect of sexual violence	Turkey	Quasi experimental	58 participants	psychosocial interventions	Effectively improve knowledge, quality of life, and reduce impact of sexual violence
3.	[26]	Effect intervention on nighttime sleep symptoms, PTSD symptoms, functional impairment, and quality of life	Canada	RCT	42 respondents	Imagery Rehearsal Therapy	Significantly reduce sleep symptoms, PTSD symptoms, and functional impairment, also the interventions can improve quality of life
4.	[27]	Effect the interventions on quality of life	USA	Quasy experimental	116 participants	Trauma Recovery Program	Significantly improve quality of life
5.	[10]	To improve quality of life and reduce negative impact of sexual violence	United States	RCT	743 respondents	Web-Based Sexual Violence Bystander Intervention	Effectively reduce negative impact on victims of sexual violence
6.	[28]	To decrease incidence and impact of sexual violence	Germany	RCT	47 children	narrative exposure therapy	Significantly reduce incidence and impact of sexual violence
7.	[9]	Effect the interventions on reduce traumatic experience	Indonesia	Quasi experiment	55 respondents	Cognitive Behaviour Therapy	Effectively reduce the trauma experienced on victim of sexual violence
8.	[29]	To reduce negative impact of sexual violence	Australia	RCT	35 students	Common Elements Treatment Approach	Effectively reduce negative impact and improve quality of life on victims of sexual violence
9.	[30]	Effect of web-based intervention to reduce post stress and depressive	USA	RCT	1250 respondents	web-based weWomen/ourCircle intervention	Significantly reduce post stress and depressive symptoms
10.	[31]	To evaluated the interventions on post-traumatic stress, traumatic grief, and anxiety symptoms	Iraq	RCT	159 respondents	Trauma-Informed Support, Skills, and Psychoeducation Intervention	Significantly reduce the traumatic experience, post traumatic stress, and anxiety symptoms

## Data Availability

Not applicable.
